# Endoscopic Papillary Large Balloon Dilation Reduces the Need for Mechanical Lithotripsy in Patients with Large Bile Duct Stones: A Systematic Review and Meta-Analysis

**DOI:** 10.1155/2014/309618

**Published:** 2014-03-06

**Authors:** Mohammad F. Madhoun, Sachin Wani, Sam Hong, William M. Tierney, John T. Maple

**Affiliations:** ^1^Division of Digestive Diseases and Nutrition, University of Oklahoma Health Sciences Center, 920 Stanton L. Young Boulevard, WP1345, Oklahoma City, OK 73104, USA; ^2^Division of Gastroenterology and Hepatology, University of Colorado, Anschutz Medical Campus, and Veterans Affairs Medical Center, Aurora, Denver, CO, USA

## Abstract

*Background*. Removal of large stones can be challenging and frequently requires the use of mechanical lithotripsy (ML). Endoscopic papillary large balloon dilation (EPLBD) following endoscopic sphincterotomy (ES) is a technique that appears to be safe and effective. However, data comparing ES + EPLBD with ES alone have not conclusively shown superiority of either technique. *Objective*. To assess comparative efficacies and rate of adverse events of these methods. *Method*. Studies were identified by searching nine medical databases for reports published between 1994 and 2013, using a reproducible search strategy. Only studies comparing ES and ES + EPLBD with regard to large bile duct stone extraction were included. Pooling was conducted by both fixed-effects and random-effects models. Risk ratio (RR) estimates with 95% confidence interval (CI) were calculated. *Results*. Seven studies (involving 902 patients) met the inclusion criteria; 3 of 7 studies were prospective trials. Of the 902 patients, 463 were in the ES + EPLBD group, whereas 439 underwent ES alone. There were no differences noted between the groups with regard to overall stone clearance (98% versus 95%, RR ** **= ** **1.01 [0.97, 1.05]; *P*  = 0.60) and stone clearance at the 1st session (87% versus 79%, RR = 1.11 [0.98, 1.25]; *P*  = 0.11). ES + EPLBD was associated with a reduced need for ML compared to ES alone (15% versus 32%; RR** ** =** ** 0.49 [0.32, 0.74]; *P*  =  0.0008) and was also associated with a reduction in the overall rate of adverse events (11% versus 18%; RR = 0.58 [0.41, 0.81]; *P*  = 0.001). *Conclusions*. ES + EPLBD has similar efficacy to ES alone while significantly reducing the need for ML. Further, ES + EPLBD appears to be safe, with a lower rate of adverse events than traditional ES. ES + EPLBD should be considered as a first-line technique in the management of large bile duct stones.

## 1. Introduction

Endoscopic retrograde cholangiopancreatography (ERCP) with endoscopic sphincterotomy (ES) represents the standard of care for management of bile duct stones [1]. However, removal of stones > 10 mm in diameter can be challenging and often requires the use of mechanical lithotripsy (ML) [2, 3]. Dilation of the biliary orifice and distal common bile duct (CBD) after ES using 12- to 20 mm esophageal or pyloric-type balloons was first described in 2003 as an alternative technique to manage large bile duct stones [4]. The safety and efficacy of this technique, termed endoscopic papillary large balloon dilation (EPLBD), have been confirmed in a number of subsequent reports [5–7]. However, studies comparing ES + EPLBD versus ES alone have not conclusively shown the superiority of either technique.

The aims of this systematic review and meta-analysis were to compare ES + EPLBD with ES alone for (i) overall clearance of stone, (ii) clearance of stones at first session, (iii) need for ML, and (iv) rate of adverse events.

## 2. Methods

### 2.1. Study Identification

All published studies that compared ES alone versus ES + EPLBD in the management of large bile duct stones were reviewed. Studies were identified by searching nine medical databases including PubMed and Ovid MEDLINE for reports published between 1994 and August of 2013. A reproducible search strategy was employed which combined the terms: “sphincterotomy” OR “ES” and “balloon dilation” and “bile duct stone” OR “choledocholithiasis. ” References from retrieved articles and abstracts presented at Digestive Diseases Week between 2003 and 2013 were also manually reviewed.

### 2.2. Study Eligibility

Two investigators (SW, SH) independently evaluated studies for inclusion in the systematic review, and any disagreements were adjudicated by the senior investigator (JM). Investigators were not blinded to journal titles, author names, or institutional affiliations. The studies (1) prospectively or retrospectively included comparative analyses between ES and ES + EPLBD, (2) reported (or provided data allowing calculation of) the overall clearance of bile duct stones, and (3) used a balloon diameter ≥ 12 mm for the ES + EPLBD arm.

### 2.3. Data Extraction

Two study investigators extracted these data independently for each study: (1) publication year, (2) country of origin, (3) study design, (4) patient demographics, (5) mean CBD diameter, (6) mean diameter of bile duct stone, (7) mean number of stones, (8) presence of periampullary diverticulum, (8) size of endoscopic sphincterotomy, (9) presence of distal common bile duct stricture, (10) use of precut sphincterotomy, (11) stone clearance at first session, (12) overall stone clearance, (13) need for mechanical lithotripsy, (14) need for extracorporeal shock wave lithotripsy, (15) mean number of sessions to achieve complete stone clearance, (16) total procedure time, (17) fluoroscopy time, and (18) rate of adverse events.

### 2.4. Outcomes for Analysis

The primary outcome of this systematic review and meta-analysis was to compare ES + EPLBD with ES alone for overall clearance of bile duct stones. Secondary outcomes included clearance of the stones at the first session, need for ML, and rate of adverse events.

### 2.5. Assessment of Study Quality

The Newcastle-Ottawa quality assessment scale was used to assess bias in studies included in this review [8]. This scale rates studies on three sources of bias based on eight criteria: (1) is the case definition adequate?; (2) representativeness of the cases; (3) selection of controls; (4) definition of controls; (5) comparability of cases and controls on the basis of the design or analysis (confounding); (6) ascertainment of exposure; (7) same method of ascertainment for cases and controls; (8) nonresponse rate. Each criterion is worth one star except confounding, which is worth two stars. The Cochrane Collaboration's Risk of Bias (a tool available in Review Manager 5) was used to assess bias in randomized trials meeting eligibility criteria [9]. This tool rates studies on four sources of bias based on six criteria: (1) adequate sequence generation to gauge selection bias; (2) allocation concealment to gauge selection bias; (3) blinding of participants, personnel, and outcome assessors to gauge performance and detection bias; (4) incomplete outcome data to gauge attrition bias; (5) selective reporting to gauge reporting bias; (6) a criterion for other forms of bias. Disagreement between the two extracting authors was resolved by consensus.

### 2.6. Data Synthesis and Statistical Analysis

The meta-analysis was performed using the Review Manager (RevMan) software, version 4.2.8 (The Nordic Cochrane Centre, Copenhagen, The Cochrane Collaboration) as a summary risk ratio (RR) with 95 percent confidence interval by using the Mantel-Haenszel fixed-effects method [10]. Estimates were also combined using the random-effects model by DerSimonian and Laird [11]. In the absence of significant heterogeneity (*P* > 0.1), the fixed-effects model results were presented. All pooled data were reported with the associated 95% confidence intervals. All statistical tests were 2 sided, and the significance level was set at 5%. Heterogeneity was assessed by both *χ* ^2^ and *I* ^2^ statistics [12, 13]. A *P* value <0.10 (or a large *χ* ^2^ statistic relative to degrees of freedom) was considered evidence of heterogeneity beyond chance. An *I* ^2^ value greater than 40% was considered substantial heterogeneity.

## 3. Results

### 3.1. Study Identifications and Selection

**Figure 1:**
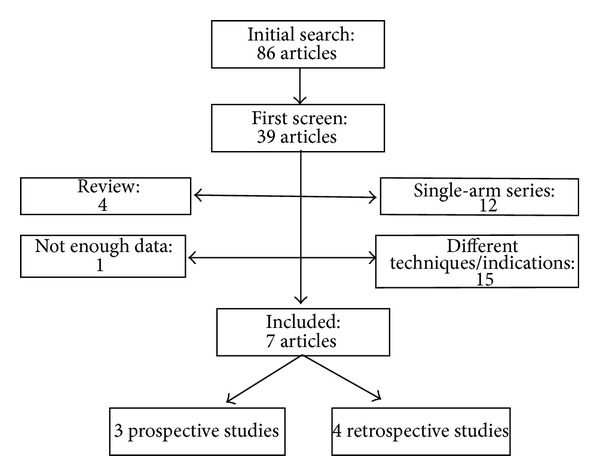
Flowchart of the studies included in the meta-analysis.

The literature search yielded 39 potentially pertinent studies for inclusion. Thirty-one of these studies were immediately excluded after initial review, most commonly because they presented a case series describing one technique or were review articles. One study met the inclusion criteria but was subsequently excluded as it described the overall rate of clearance among the ES + EPLBD group but not among the ES group [14]. Thus seven studies were included in the final analysis (Figure 1) [15–21]. Manual review of the references of retrieved manuscripts concerning ES + EPLBD versus ES alone did not yield any additional studies meeting inclusion criteria for this analysis.

### 3.2. Description of Variation in Study Methods

Of the seven studies meeting inclusion criteria for the meta-analysis (Table 1), three were prospective randomized studies [15, 17, 20] and four were retrospective studies [16, 18, 19, 21]. One study was published as an abstract [18] and the rest were complete papers [15–17, 19–21]. Four studies were conducted in Korea [15, 17–19], one in Japan [16], one in China [20], and one in Portugal [21]. All studies except one [18] provided detailed patient demographic information. The number of patients included in these studies ranged from 27 to 100. The range of balloon diameter used in the ES + EPLBD group was between 12 and 20 mm in the six studies that reported this variable. Four of these studies used balloons with a minimum diameter of 15 mm [16–18, 20], while two studies used balloons ≥ 12 mm [15, 21]. No study specified the exact distribution of balloon diameters used within the range of permissible balloon sizes. A limited (submaximal) ES was implemented in conjunction with EPLBD in four studies [15, 17, 19, 20], full/maximal ES in one study [16], and two studies did not mention the extent of ES [18, 21]. All studies reported rates for overall stone clearance and the use of mechanical lithotripsy, and all except one [18] reported rates of clearance at the first session. Three studies reported total procedure time [16, 19, 20], and one study reported fluoroscopy time [16]. Five studies reported data regarding the size and/or number of biliary stones [15–17, 20, 21]. Among the three prospective randomized trials, randomization was conducted intraprocedurally after obtaining biliary access in two studies [15, 20] and prior to ERCP in one study [17].

### 3.3. Assessment of Study Quality

**Figure 2:**
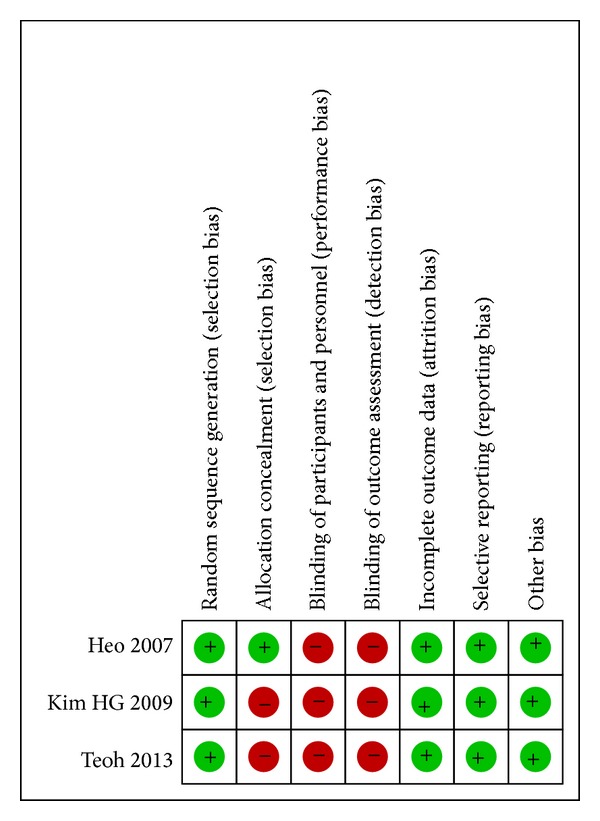
Risk of bias summary of randomized clinical trials.

The assessment of bias in the retrospective studies indicated that three studies were of high methodological quality [16, 19, 21] and one of moderate methodological quality (Table 2) [18]. There appeared to be a consistent performance bias among all three randomized trials because of the fact that the nature of the study precludes blinding of the operator. Two studies were also limited by selection bias because of inadequate allocation concealment (Figure 2) [19, 20].

### 3.4. Data Synthesis

Seven studies involving 902 patients met the inclusion criteria. Of the 902 patients, 439 subjects were in the ES group, while 463 subjects underwent ES + EPLBD. The median stone diameter in the ES + EPLBD group was 16 mm compared to 15.3 mm in the ES group. The between-study variability (i.e., heterogeneity) beyond what could be expected by sampling error was substantially high for the pooled RR of overall clearance rate, clearance at 1st session, and the use of mechanical lithotripsy; hence, the random effects model was used. The *I* ^2^ of the pooled RR of the overall complication rate was 0%, which was not considered substantial heterogeneity as per* a priori* definition and thus the fixed effects model was used.

#### 3.4.1. Overall Stone Clearance and Stone Clearance at 1st Session

**Figure 3:**
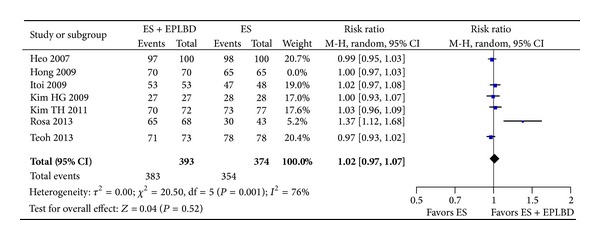
Forrest plot of the pooled risk ratio of overall clearance of duct stones and *I* ^2^ statistic for heterogeneity.

**Figure 4:**
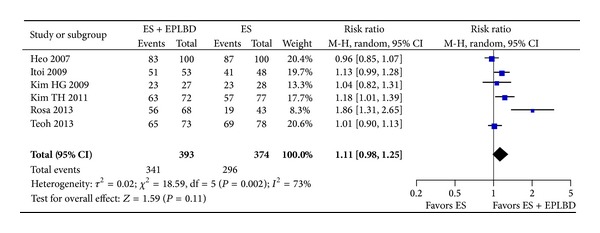
Forrest plot of the pooled risk ratio of clearance of stone at 1st session and *I* ^2^ statistic for heterogeneity.

The rate of overall stone clearance was not significantly different between the ES + EPLBD group and the ES group (98% versus 95%, RR* * = * *1.01 [0.97, 1.05]; *P*  =  0.60) (Figure 3). Similarly, the rate of stone clearance at the 1st session was not significantly different between the ES + EPLBD group and the ES group (87% versus 79%, RR = 1.11 [0.98, 1.25]; *P* = 0.11) (Figure 4).

#### 3.4.2. Number of Sessions, Need for Mechanical Lithotripsy, and Procedure Duration

**Figure 5:**
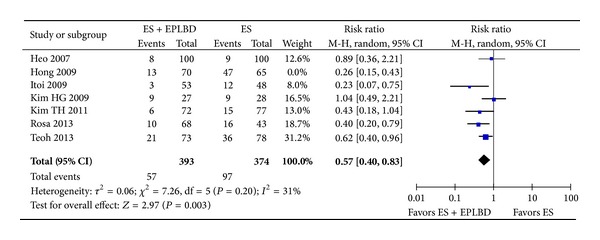
Forrest plot of the pooled risk ratio of the use of mechanical lithotripsy and *I* ^2^ statistic for heterogeneity.

Among the four studies [17–19, 21] that reported the mean number of sessions needed for complete stone clearance, there was a nonsignificant trend toward fewer sessions needed among those who underwent ES + EPLBD versus ES (1.3 ± 0.3 versus 1.8 ± 0.7, *P* = 0.245). ES + EPLBD was associated with a reduced need for ML compared to ES alone (15% versus 32%; RR * *= * *0.49 [0.32, 0.74]; *P*  =  0.0008) (Figure 5). Total procedure time was numerically shorter in the ES + EPLBD group in each of the three studies that reported this variable [16, 17, 20], though this was statistically significant in only one study [16]. Total fluoroscopy time was significantly shorter in the ES + EPLBD group in the single study that reported this variable [16].

#### 3.4.3. Adverse Events

**Figure 6:**
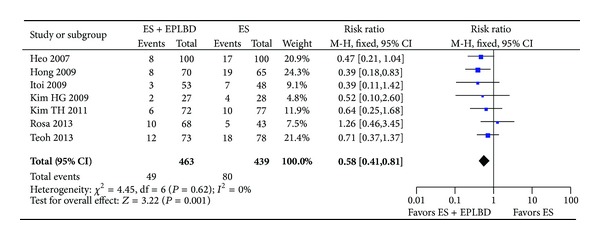
Forrest plot of the pooled risk ratio of the overall rate of adverse events and *I* ^2^ statistic for heterogeneity.

ES + EPLBD was associated with a reduction in the overall rate of adverse events compared to ES alone (11% versus 18%; RR = 0.58 [0.41, 0.81]; *P* = 0.001) (Figure 6). A subgroup analysis showed a significantly lower overall bleeding rate in the ES + EPLBD group (4% versus 9%; RR = 0.5 [0.3, 0.8]; *P* = 0.003). Bleeding could be further stratified into intraprocedural bleeding and clinically significant bleeding. The rate of intraprocedural bleeding was significantly higher in the ES alone group (8% versus 4%, *P* = 0.008), and while the rate of clinically significant bleeding was also numerically higher in the ES alone group, this trend did not attain statistical significance (0.9% versus 0.3%, *P* = 0.19). Individual studies generally required a 2-3 gram decrease in hemoglobin and/or overt melena for bleeding to be classified as “clinically significant.” The rates of pancreatitis, perforation, and cholangitis were similar between the two groups (Table 3). No instances of perforation occurred in the 463 patients undergoing ES + EPLBD. Three perforations occurred in the ES arm: one retroperitoneal perforation of a diverticular wall during ES [19] and two suspected guidewire perforations [20]. All three perforations were managed conservatively. With regard to pancreatitis, a subanalysis demonstrated no difference in the rate of severe pancreatitis between patients undergoing ES versus ES + EPLBD (1.2% versus 0.7%, *P* = 0.7) [15, 17, 20, 21]. No life-threatening complications were reported across all patients in both groups.

#### 3.4.4. Sensitivity Analyses

**Figure 7:**
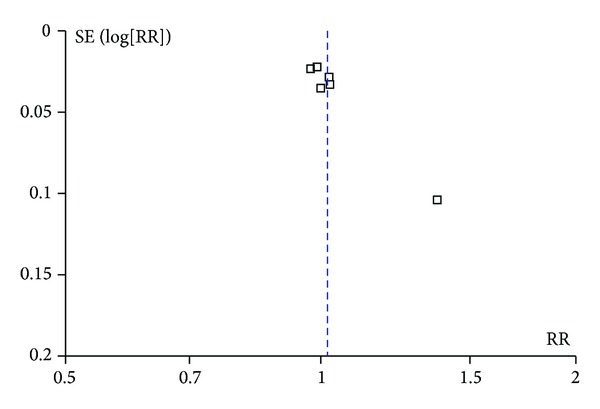
Funnel plot of the risk ratio of the overall clearance of bile duct stones.

Sensitivity analyses were performed and most of the heterogeneity observed for the calculation of pooled overall clearance rate and clearance at 1st session could be attributed to a single study [21]. However, removing this study from the analysis did not alter the results. Similarly, no significant changes were seen across all outcomes when the study published only in abstract form was removed [18]. The retrospective and prospective studies were also analyzed separately. The pooled RR estimates for all primary and secondary outcomes when analyzed separately for only retrospective studies and for only prospective studies were concordant with pooled RR estimates for all of the studies combined (Table 4). A funnel plot of the overall clearance rate showed some asymmetry due to an unusually low rate of overall clearance (70%) in the ES-only arm in the study conducted by Rosa et al. (see Figure 7) [21].

## 4. Discussion

Approximately 85%–90% of bile duct stones can be removed with a balloon or basket following ES, with the most common reason for failure being large stone size [2, 22]. Historically, stones > 10 mm in diameter, and especially stones > 15 mm in diameter, have been associated with a lower success rate for endoscopic removal and a more frequent need for lithotripsy [3, 23]. Beyond stone size, factors such as small diameter of the distal CBD, ductal strictures distal to stones, and inadequate sphincterotomy, all may negatively impact the success rate of stone extraction at ERCP.

Despite the fact that the studies populating this meta-analysis were enriched with patients harboring large bile duct stones, we observed very high rates of success for overall duct clearance for both EPLBD after ES (98%) and for ES alone (95%). This likely reflects the expertise of the centers conducting these trials. Nonetheless, there was a trend observed towards more frequent clearance of the duct in one session with EPLBD after ES (87%) as compared with ES alone (79%, RR = 1.11 [0.98, 1.25]; *P* = 0.11). As such, it remains possible that rates of single session duct clearance may be enhanced with EPLBD after ES.

While mechanical lithotripsy is a valuable tool in the management of large or difficult stones, there are also potential issues associated with this technique including the challenge of cannulation with a through-the-scope lithotripter, difficulty with stone capture, need for repeated duct sweeping to remove stone fragments, and the potential need for stent placement if adequate duct clearance is not attained. EPLBD after ES holds potential advantages over ML in that many of these issues are avoided, as cannulation with the dilating balloon is not difficult, distal obstructive phenomena (e.g., duct strictures and inadequate sphincterotomies) are treated, and stones are removed* in toto*. As a result, stone clearance might be accomplished more quickly with this technique.

In this meta-analysis we found a significant reduction in the need for ML when EPLBD was employed after ES (15%) as compared with ES alone (32%). Limitations in data reporting preclude firm conclusions regarding time savings, but trends were noted for reductions in total procedure time and fluoroscopy time in the ES + EPLBD group when these variables were reported.

The use of EPLBD after ES was also associated with a reduction in the overall rate of adverse events as compared with ES alone. However, at least some of these differences appeared to be due to differences in intraprocedural bleeding events. Establishing a consistent threshold for reporting this endpoint is difficult, and for the endpoint of clinically significant bleeding, which may be more relevant, there was not a significant difference between the two groups. The rates for pancreatitis and cholangitis were similar between the two groups, and there were no perforations in the 463 patients who underwent ES + EPLBD. As such, it appears that ES + EPLBD is at least as safe as, and may be safer than, ES alone in patients undergoing ERCP for large bile duct stones.

A recently published meta-analysis [24] similarly aimed to compare ES alone versus ES + EPLBD. However, this meta-analysis is hindered by significant flaws with regard to study eligibility, inclusion criteria, and quality assessment that collectively detract from its overall validity. In this analysis, the authors erroneously included one study which was a randomized clinical trial of ES versus primary balloon sphincteroplasty (no preceding ES) using smaller balloons that did not exceed 12 mm in diameter [25]. The analysis also inappropriately included a randomized trial that compared ES + EPLBD versus ES + mechanical lithotripsy; in this study, no patients who underwent ES + EPLBD could receive ML, whereas all patients in the ES arm underwent ML also [26]. Another methodological concern involves the use of the Jadad score (an assessment tool only validated for assessing the quality of randomized clinical trials) to evaluate two retrospective studies [16, 19]. Finally, the authors reported that five studies in their analysis were “double blinded”—an impossibility given the nature of the interventions. These shortcomings grossly invalidate the findings of this analysis and the data we present represents the first methodologically robust meta-analysis comparing these two techniques.

The pooled results from this analysis are consistent with the trend from the majority of the included studies, which lacked adequate sample size to reach statistical significance independently. The results of this meta-analysis appear generalizable, given the variety of clinical settings, patient diversity, relatively uniform, readily available devices, and simple technique used to perform EPLBD after ES.

There are limitations in this study that merit mention. The inclusion of only English language studies could have potentially excluded some relevant trials. Significant heterogeneity noticed between the studies may be related to variations in study design, participant selection, use of different balloon sizes for dilation, the extent of the sphincterotomy, and operator skill level with mechanical lithotripsy. However, a sensitivity analysis that removed one study at a time demonstrated no effect on the overall conclusion of the pooled analysis. Combining retrospective and prospective studies can be problematic and is only advisable if the segregated results of pooled analyses of the retrospective and prospective studies are consistent, which is the case in our analysis.

In conclusion, the available literature demonstrates that ES + EPLBD has similar efficacy to ES alone for the removal of large bile duct stones while significantly reducing the need for ML. Further, ES + EPLBD appears to be safe, with a lower overall rate of complications in this pooled analysis relative to ES alone. ES + EPLBD should be considered a first-line technique in the management of large bile duct stones. Future prospective studies may allow for better comparisons between the two techniques with regard to total procedure time, fluoroscopy time, and costs.

## Figures and Tables

**Table 1:** Characteristics of included studies.

Study (year)	Country	Type of study	Number of patients ES/ES + EPLBD	Age (mean) ES/ES + EPLBD	Gender, M (%) ES/ES + EPLBD	Mean CBD diameter (mm ± SD) ES/ES + EPLBD	Large balloon dilation size (mm)	Mean size of the stone (mm ± SD) ES/ES + EPLBD	Size of ES ES/ES + EPLBD	Periampullary diverticulum (%) ES/ES + EPLBD
Heo (2007)	Korea	RCT	100/100	63/64	50/48	N/A	12–20	15 ± 0.7/16 ± 0.7	Full/Limited	45/49
Itoi (2009)	Japan	Retrospective	48/53	73/75	58/38	18 ± 4.3/17 ± 3.7	15–20	15 ± 3.2/15 ± 3.5	Full/Full	58/47
Kim HG (2009)	Korea	RCT	28/27	70/70	39/37	21 ± 5.7/21 ± 6.3	15–18	21 ± 5.2/21 ± 4.1	Full/Limited	36/33
Hong GY (2009)	Korea	Retrospective	65/70	N/A	N/A	N/A	15 or 20	N/A	N/A	49/57
Kim TH (2011)	Korea	Retrospective	77/72	69/69	49/54	19 ± 4.4/18 ± 3.3	N/A	N/A	Full/Limited	N/A
Teoh (2013)	China	RCT	78/73	73/72	51/44	N/A	15	N/A	Full/Limited	NA
Rosa (2013)	Portugal	Retrospective	43/68	73/71	35/34	16.4 ± 7.2/17.1 ± 3.4	12–18	16.0 ± 6.7/16.8 ± 4.4	N/A	N/A

RCT: randomized controlled trial; ES: endoscopic sphincterotomy; EPLBD: endoscopic papillary large balloon dilation; N/A: not available; CBD: common bile duct; SD: standard deviation.

**Table 2:** The Newcastle-Ottawa quality assessment in retrospective studies.

Study (year)	Selection	Comparability	Outcome/exposure
Itoi (2009)	∗∗∗∗	∗∗	∗∗
Hong GY (2009)	∗∗∗	N/A	∗∗
Kim TH (2011)	∗∗∗∗	∗∗	∗∗
Rosa (2013)	∗∗∗∗	∗∗	∗∗

**Table 3:** Subgroup analysis of the complications rate.

	ES	ES + EPLBD	RR, (95% CI)	*P* value
All bleeding	38/439	20/463	0.5 (0.3, 0.8)	0.01
Pancreatitis	29/439	23/463	0.8 (0.4, 1.3)	0.29
Perforation	3/439	0/463	Not estimable	
Cholangitis	4/439	4/463	1.0 (0.24, 3.77)	0.94

**Table 4:** Sensitivity analysis of the primary and secondary outcomes stratified by the type of the studies.

		Relative risk (EPLBD + ES versus ES), (95% CI)	
	Retrospective studies	Randomized clinical trials	Combined
Overall clearance of bile duct stone	1.06 (0.95, 1.01)	0.98 (0.97, 1.01)	1.01 (0.97, 1.05)
Clearance of the stones at 1st session	1.29 (1.01, 1.46)	0.99 (0.91, 1.07)	1.11 (0.98, 1.25)
Need for ML	0.31 (0.22, 0.44)	0.73 (0.52, 1.03)	0.49 (0.32, 0.74)
Overall complication rate	0.57 (0.36, 0.91)	0.59 (0.36, 0.95)	0.58 (0.41, 0.81)
